# Factors associated with early 14-day unplanned hospital readmission: a matched case–control study

**DOI:** 10.1186/s12913-021-06902-6

**Published:** 2021-08-25

**Authors:** Yu-Tai Lo, Chia-Ming Chang, Mei-Hua Chen, Fang-Wen Hu, Feng-Hwa Lu

**Affiliations:** 1 Department of Geriatrics and Gerontology, National Cheng Kung University Hospital, College of Medicine, National Cheng Kung University, No. 138, Sheng Li Road, 70403 Tainan, Taiwan; 2 Department of Medicine, Institute of Gerontology, College of Medicine, National Cheng Kung University, Tainan, Taiwan; 3 Department of Internal Medicine, National Cheng Kung University Hospital, College of Medicine, National Cheng Kung University, Tainan, Taiwan; 4 Department of Nursing, National Cheng Kung University Hospital, Tainan, Taiwan; 5 Department of Nursing, College of Medicine, National Cheng Kung University, Tainan, Taiwan; 6 Division of Geriatric Medicine, Department of Medicine, College of Medicine, National Cheng Kung University, Tainan, Taiwan

**Keywords:** Patient Discharge, Patient Readmission, Quality Indicators, Risk Factors

## Abstract

**Background/Purpose:**

Early unplanned hospital readmissions are burdensome health care events and indicate low care quality. Identifying at-risk patients enables timely intervention. This study identified predictors for 14-day unplanned readmission.

**Methods:**

We conducted a retrospective, matched, case–control study between September 1, 2018, and August 31, 2019, in an 1193-bed university hospital. Adult patients aged ≥ 20 years and readmitted for the same or related diagnosis within 14 days of discharge after initial admission (index admission) were included as cases. Cases were 1:1 matched for the disease-related group at index admission, age, and discharge date to controls. Variables were extracted from the hospital’s electronic health records.

**Results:**

In total, 300 cases and 300 controls were analyzed. Six factors were independently associated with unplanned readmission within 14 days: previous admissions within 6 months (OR = 3.09; 95 % CI = 1.79–5.34, *p* < 0.001), number of diagnoses in the past year (OR = 1.07; 95 % CI = 1.01–1.13, *p* = 0.019), Malnutrition Universal Screening Tool score (OR = 1.46; 95 % CI = 1.04–2.05, *p* = 0.03), systolic blood pressure (OR = 0.98; 95 % CI = 0.97–0.99, *p* = 0.01) and ear temperature within 24 h before discharge (OR = 2.49; 95 % CI = 1.34–4.64, *p* = 0.004), and discharge with a nasogastric tube (OR = 0.13; 95 % CI = 0.03–0.60, *p* = 0.009).

**Conclusions:**

Factors presented at admission (frequent prior hospitalizations, multimorbidity, and malnutrition) along with factors presented at discharge (clinical instability and the absence of a nasogastric tube) were associated with increased risk of early 14-day unplanned readmission.

## Introduction

Hospital readmissions disrupt the normal lives of families and caregivers, cause patient discomfort, and increase overall health care costs [1–3]. Hospital readmission rate is also considered a performance indicator for measuring a hospital’s quality of care [4]. Recent policies on readmission have imposed financial penalties; for instance, the US Centers for Medicare & Medicaid Services reduced reimbursements to hospitals with high 30-day risk-standardized all-cause readmission rates. In England and Germany, any readmission occurring within 30 days from discharge from an elective admission is no longer reimbursed [5]. Therefore, preventing unnecessary hospital readmissions can potentially both reduce financial health care burdens and improve the quality of care [6, 7].

An approach to reducing the hospital readmission rate is identifying patients at risk of readmission, which allows for further investigation and development of preventive strategies because many readmissions are considered preventable [8, 9]. Nevertheless, diverse and complex factors lead to readmissions, and clinicians are unable to process information to accurately identify at-risk patients [10]. Studies have suggested various risk factors for 30-day readmission, including age, social determinants, Charlson Comorbidity Index, prior health care utilization patterns, emergent admission, laboratory data including hemoglobin and sodium levels, discharge from an oncological service, procedures during the index admission (first admission), and length of hospital stay [11, 12].

Compared with planned readmission, unplanned readmission is more representative of substandard care during the initial admission. The likelihood of unplanned readmissions is the highest in the immediate postdischarge period [2]. Early 14-day unplanned readmissions were demonstrated to be associated with quality of inpatient care; thus, they were deemed avoidable in cases of high-quality care [3]. Recent studies have specifically shown that early readmissions within the first 7 days of hospital discharge may be more preventable than later 30-day readmissions are [8, 13, 14], and they are more indicative of potential gaps in care during the index hospitalization [13, 15]. Furthermore, studies have also demonstrated a variation in the strength of unplanned 7-day and 30-day readmission predictors [14, 15]. However, whether risk factors of 14-day unplanned hospital readmissions vary from those of 7-day or 30-day unplanned readmissions has not been thoroughly investigated.

As a continuous monitoring indicator of care quality recommended by the National Health Insurance Administration, the target unplanned 14-day hospital readmission rate for the same or a related diagnosis was set to 5.75 % in 2020 as a national standard for all hospitals across Taiwan [16]. The rate of unplanned 14-day hospital readmission ultimately affects hospital accreditation and indirectly influences government reimbursements to hospitals in Taiwan [17]. Policymakers and health care professionals therefore should understand risk factors associated with early 14-day unplanned readmission to implement or modify measures that health care systems, reduce health care expenditures, and improve the quality of care. However, only limited descriptive studies have reported the medical factors, including deterioration of underlying diseases, recurrent medical conditions, and major diagnoses, associated with 14-day unplanned readmission in Taiwan. A retrospective matched case–control study with 83 case group and 69 control group members concluded that patients who were dependent in daily activities and had more drug prescriptions at discharge were more likely to be readmitted [18]. However, the study did not investigate the effects of previous health care utilization, malnutrition, or laboratory data. Therefore, in this study, to better understand the factors for predicting 14-day unplanned readmission in Taiwan and to clarify whether they differ from those predicting 7-day or 30-day readmission (as reported in the literature), we identified key risk factors for early unplanned readmission within 14 days after hospital discharge.

## Materials and methods

### Study design and location

We conducted a retrospective, matched, case–control study to identify risk factors for 14-day unplanned readmission. The study protocol was approved by the Institutional Review Board of the National Cheng Kung University Hospital (A-ER-108-309). The requirement for consent was waived because this was a retrospective medical record study. The study population included adult patients aged ≥ 20 years who underwent hospital discharges consecutively from September 1, 2018, through August 31, 2019, at an 1193-bed tertiary care university hospital in Tainan, Taiwan. Patients who were admitted for cancer-related treatments, participated in pharmaceutical clinical trials, were discharged against medical advice, died during admission, and lived abroad were excluded from the study.

### Case–control selection

Patients who experienced an unplanned readmission within 14 days after the index admission were included as cases. Unplanned readmission was defined as admission for the same or a related diagnosis and was confirmed through a review of patient medical records by discharge planning nurses. Patients who had been hospitalized but did not have an unplanned readmission within 14 days of discharge were included as controls. Age is a known readmission risk factor, and disease-related groups are related to resource consumption during hospitalization. Changes in staffing, facilities, clinical practice, and referral patterns over the 1-year study period may have introduced unintended bias in the study findings. Therefore, each case was matched for the same disease-related group at index admission and 1:1 propensity-score matched for age and discharge date. We excluded cases that could not be matched.

### Data source

All medical records of the hospital were computerized. The data set for this study was extracted from the medical records of the hospital.

### Study variables

Based on a literature review [11, 12], we identified unplanned readmission risk factors and factors of interest. Patient demographic characteristics included sex, marital status, religion, education level, and area of residence. Previous health care utilization factors included hospitalizations, emergency department (ED) visits, and outpatient visits 6 months prior to index admission. Factors related to comorbidity included number of diagnoses according to the three major diagnoses at outpatient services and during admission 1 year before the index admission, Charlson Comorbidity Index (CCI), and diagnoses based on CCI [19]. Type of index admission and functional evaluation upon admission, which included the ability to move without assistance, ability to bathe without assistance, nutrition status according to the Malnutrition Universal Screening Tool (MUST) [20], mood status according to the Brief Symptom Rating Scale (BSRS-5) [21], presence of incontinence, and history of fall 1 year prior to admission, were recorded. Laboratory values recorded before discharge included white blood cell (WBC) count, platelet count, hemoglobin, creatinine, alanine aminotransferase (ALT), potassium, and sodium. Discharge-related factors included whether a patient was listed in the hospital discharge planning services; vital signs (systolic blood pressure, diastolic blood pressure, pulse rate, respiratory rate, and ear temperature) recorded 24 h prior to discharge, medical department of discharge; years of experience of the attending physician; length of hospital stay; number of discharge medication categories; total number of tablets of discharge medication; average number of daily medication tablets; discharge destination; discharge with pressure injury (or injuries); and discharge with a nasogastric (NG) tube, Foley catheter, trachea tube, or any other indwelling catheter(s).

### Data validation

Data included in the analysis were validated through a review of medical records of randomly identified patient numbers.

### Statistical analysis

We compared the continuous variables and categorical variables between cases and controls, and the results are expressed as means and percentages, respectively. If normally distributed, continuous variables were assessed using Student *t* tests; otherwise, the Mann–Whitney *U* test was used to evaluate associations with unplanned readmission among the matched strata of cases and controls. For the categorical variables, the chi-squared test or Fisher’s exact test was used. Factors independently associated with 14-day unplanned readmission were identified through multivariate logistic regression. Considering the paucity of information on risk factors for 14-day unplanned readmission in Taiwan and to avoid false positives, we adopted the automatic variable selection procedure with forward selection (conditional). Variance inflation factor (< 4) was used to detect collinearity between variables in the multivariate model. Odds ratios (ORs) and 95 % confidence intervals (CI) are reported. *p* < 0.05 was considered significant for all analyses. All statistical analyses were conducted using SPSS Version 22 (IBM Corp., Armonk, NY, USA).

## Results

### Study population

**Fig. 1:**
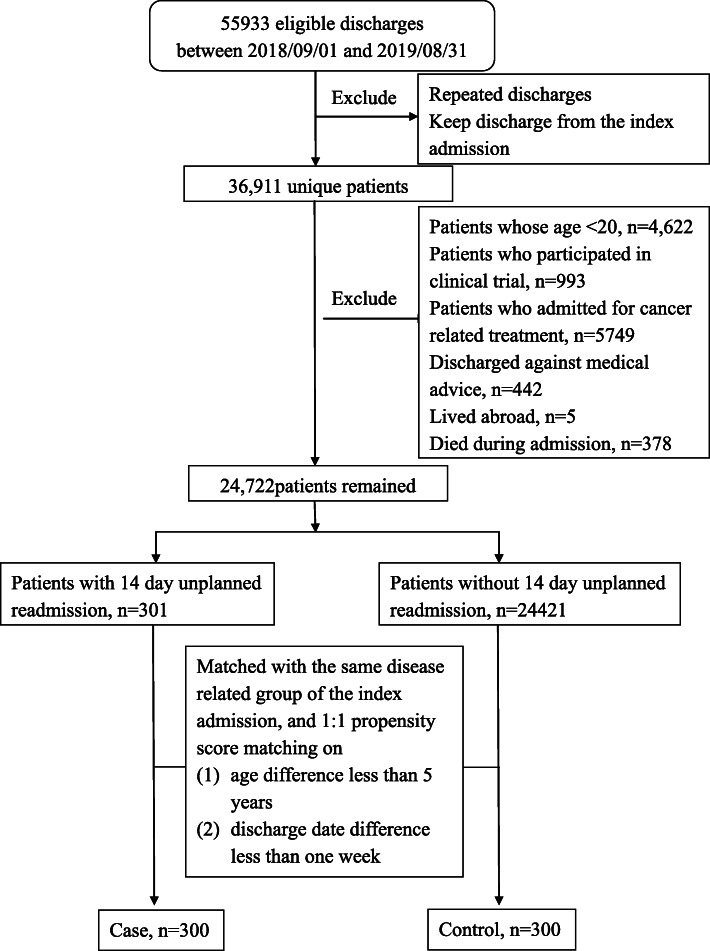
Study flowchart of the case-control selection

From September 1, 2018, through August 31, 2019, a total of 36,911 adult patients were discharged, with a total of 55,933 discharges (including repeated admissions). After exclusion, 301 adult patients with unplanned readmission (cases) and 24,421 adult patients with no unplanned readmission (unplanned readmission rate: 1.23 %) were included in the analysis. No match was obtained for one case, which was then excluded. A total of 300 cases and 300 controls were ultimately studied (Fig. 1). The 300 enrolled cases had a mean [± standard deviation (SD)] age of 65 (± 17.74) years. Among the cases, 61 % of unplanned readmissions occurred within 7 days of hospital discharge. The major reason for readmission was deterioration of existing disorder (65.5 %), followed by soft tissue infection wounds (7.0 %) and pulmonary infection (5.7 %). Reasons for unplanned readmission are listed in Table 1.

**Table 1:** Reasons for unplanned readmission (*N* = 300)

Reasons for unplanned readmission	Cases	
	N	%
Deterioration of existing disorder	196	65.5
Soft tissue infection wounds	21	7.0
Pulmonary infection	17	5.7
Complications after surgery	17	5.7
Fever of unknown origin	8	2.7
Dyspnea or respiratory distress	4	1.3
Gastrointestinal tract hemorrhage	6	2.0
Active labor	6	2.0
Urinary tract infection	4	1.3
Personal or family factors	4	1.3
Pain	3	1.0
Abnormalities of implants or devices	2	0.7
Change in consciousness	1	0.3
Complications after treatment	1	0.3
Other	10	3.3

### Univariate analysis between cases and controls

A univariate comparison of demographic characteristics, previous health care utilization, and comorbidities between cases and controls is presented in Table 2. Compared with controls, cases had significantly higher previous health care utilization including hospitalizations [0.98 (± 1.39) vs. 0.11 (± 0.47), *p* < 0.001], ED visits [1.98 (± 2.23) vs. 1.11 (± 1.47), *p* < 0.001], and outpatient visits [7.52 (± 8.17) vs. 4.78 (± 5.43), *p* < 0.001] within 6 months prior to index admission. Regarding comorbidity factors in the past year, cases demonstrated a significantly higher comorbidity burden than did controls, with significantly higher numbers of diagnoses [8.55(± 6.43) vs. 5.26 (± 5.15), *p* < 0.001] and CCI scores [5.24(± 5.66) vs. 3.12 (± 4.76), *p* < 0.001]. Cases were more likely to have received diagnoses of chronic pulmonary disease (12.7 % vs. 6.7 %, *p* = 0.013), moderate to severe liver disease (3.3 % vs. 0 %, *p* = 0.001), diabetes (16.0 % vs. 10.3 %, *p* = 0.040), and cancer (24 % vs. 14.7 %, *p* = 0.004) than controls were. No statistical differences in other variables (sex, marital status, religion, education level, area of residence, or other diseases) were observed between cases and controls.

**Table 2:** Comparisons of demographic characteristics, previous health care utilization, and comorbidities between cases and controls

Variables	Cases		Controls		*p* value
	*N* = 300		*N* = 300		
**Sociodemographic**					
Sex, female (%)	134	(44.7)	143	(47.7)	0.478
Age (years), mean (SD)	65	(17.74)	65.1	(18.31)	-
Age group, n (%)					0.945
20–49	63	(21.0)	66	(22.0)	
50–64	72	(24.0)	64	(21.3)	
65–74	58	(19.3)	57	(19.0)	
75–84	64	(21.3)	69	(23.0)	
≥85	43	(14.3)	44	(14.7)	
Marital status, single (%)	79	(26.3)	93	(31.0)	0.338
Religion, yes (%)	231	(77.0)	246	(82.0)	0.119
Education, ≤elementary school (%)	127	(43.2)	135	(45.9)	0.507
Area of residence, urban (%)	240	(80.0)	235	(78.3)	0.671
**Health care utilization in the past 6 months**					
No. of outpatient visits, mean (SD)	7.52	(8.17)	235	(5.43)	<0.001
No. of hospitalizations, mean (SD)	0.98	(1.39)	0.11	(0.47)	<0.001
No. of emergency visits, mean (SD)	1.98	(2.23)	1.11	(1.17)	<0.001
**Comorbidities in the past 1 year**					
No. of diagnoses, mean (SD)	8.55	(6.43)	5.26	(5.15)	<0.001
Charlson Comorbidity Index, mean (SD)	5.24	(5.66)	3.12	(4.76)	<0.001
Diseases, n (%)					
Cancer	72	(24.0)	44	(14.7)	0.004
Moderate to severe renal disease	68	(22.7)	58	(19.3)	0.316
Diabetes	48	(16.0)	31	(10.3)	0.040
Diabetes with end organ damage	45	(15.0)	42	(14.0)	0.728
Chronic pulmonary diseases	38	(12.7)	20	(6.7)	0.013
Congestive heart failure	22	(7.3)	18	(6.0)	0.513
Cerebrovascular diseases	21	(7.0)	19	(6.3)	0.743
Mild liver disease	19	(6.3)	10	(3.3)	0.087
Peptic ulcer disease	17	(5.7)	19	(6.3)	0.731
Dementia	15	(5.0)	14	(4.7)	0.849
Myocardial infarction	12	(4.0)	5	(1.7)	0.085
Moderate to severe liver disease	10	(3.3)	0	0	0.001
Connective tissue diseases	8	(2.7)	4	(1.3)	0.243
Metastatic cancer	8	(2.7)	3	(1.0)	0.128
Peripheral vascular diseases	7	(2.3)	2	(0.7)	0.176

*SD* standard deviation

The univariate comparisons of type of index admission, functional evaluation, and laboratory data between cases and controls are presented in Table 3. Cases were more likely to be admitted from emergency room (74 % vs. 63 %, *p* = 0.001), were less independent in terms of mobility (36.3 % vs. 44.7 %, *p* = 0.008) and bathing (52.3 % vs. 63.3 %, *p* = 0.048), had a higher risk of malnutrition [average scores of MUST: 0.67 (± 0.94) vs. 0.52 (± 0.97), *p* = 0.045], had a greater proportion of incontinence (27.3 % vs. 20.7 %, *p* = 0.040), and had a lower hemoglobin level [11.22 (± 2.09) vs. 11.73 (± 2.40), *p* < 0.001]. No statistical differences in any other variables (mood, falls in the past 1 year, WBC count, platelet count, Creatinine, ALT, potassium, and sodium) were observed between cases and controls.

**Table 3:** Comparisons of clinical conditions upon admission, and laboratory data between cases and controls

Variables	Cases		Controls		*p* value
	*N* = 300		*N* = 300		
**Admission via emergency room (%)**	222	(74.0)	189	(63.0)	0.001
**Clinical conditions upon admission**					
Mobility, independent (%)	109	(36.3)	134	(44.7)	0.008
Bathing, independent (%)	157	(52.3)	190	(63.3)	0.048
Nutrition (MUST), mean (SD)	0.67	(1.10)	0.52	(0.97)	0.045
MUST, low risk: 0 (%)	195	(66.3)	215	(72.6)	0.225
MUST, medium risk: 1 (%)	37	(12.6)	33	(11.1)	
MUST, high risk: 2–4 (%)	62	(21.1)	48	(16.2)	
Mood (BSRS), mean (SD)	0.67	(0.94)	0.78	(1.11)	0.257
Urine incontinence, yes (%)	82	(27.3)	62	(20.7)	0.040
Fall in the past 1 year, yes (%)	52	(17.3)	40	(13.3)	0.193
**Laboratory data, mean (SD)**					
White blood cell, ×10^3^ /UL	8.38	(3.77)	8.01	(3.47)	0.183
Hemoglobin, g/dL	11.22	(2.09)	11.73	(2.40)	0.001
Platelet, ×10^3^ /UL	229.34	(103.18)	234.25	(104.55)	0.567
Creatinine, mg/dL	1.64	(2.33)	1.44	(1.83)	0.243
Alanine aminotransferase, U/L	37.95	(71.30)	32.78	(51.86)	0.297
Potassium, mmol/L	3.90	(0.46)	4.01	(2.23)	0.423
Sodium, mmol/L	135.76	(11.59)	137.31	(8.02)	0.076

*SD* standard deviation, *BSRS* Brief Symptom Rating Scale, *MUST* Malnutrition Universal Screening Tool

The comparisons of discharge-related factors between cases and controls are presented in Table 4. A higher proportion of cases received discharge care planning (40 % vs. 32 %, *p* = 0.032), had a longer hospital stay [11.24 (± 13.13) days vs. 9.14 (± 10.17 days), *p* = 0.005], and were more likely to have pressure injury (injuries) (23.3 % vs. 14.7 %, *p* = 0.004) upon discharge. Moreover, cases had a higher number of catheters at discharge [0.80 (± 0.06) vs. 0.64 (± 0.05), *p* = 0.005] than did controls. Regarding vital signs 24 h before discharge, cases presented with higher pulse rate [83.26 (± 14.18) per min vs. 80.39 (± 12.92) per min, *p* = 0.008] and respiratory rate [18.41 (± 2.29) per min vs. 18.08 (± 1.79) per min, *p* = 0.03], but a lower proportion of systolic blood pressure over 100 mmHg before discharge (94.0 % vs. 97.7 %, *p* = 0.025). Cases were prescribed more discharge medication than were controls in terms of total number of categories [6.99 (± 4.36) vs. 5.60 (± 3.99), *p* < 0.001], total number of tablets [77.14 (± 56.00) vs. 63.26 (± 60.52), *p* = 0.002], and average number of daily tablets [11.27 (± 7.64) vs. 9.27 (± 6.83), *p* < 0.001]. No significant differences in any other variables (medical department of discharge, physician’s experience in years, destination of discharge, diastolic blood pressure, ear temperature, and the type of catheter(s) upon discharge) were observed between the case and control groups.

**Table 4:** Comparisons of related factors upon discharge between cases and controls

Variables	Cases		Controls		*p* value
	*N* = 300		*N* = 300		
**Discharge planning services**, yes (%)	120	(40.0)	96	(32.0)	0.032
**Medical department of discharge**					0.796
Internal medicine	180	(60.0)	172	(57.3)	
Surgical department	63	(21.0)	69	(23.0)	
Obstetrics and gynecology	13	(4.3)	12	(4.0)	
Others	44	(14.6)	47	(15.7)	
**Physician’s experience (years)**, mean (SD)	8.55	(7.34)	8.46	(7.45)	0.886
**Destination of discharge**					-
Home and outpatient follow-up	292	(97.3)	290	(96.7)	
Admitted to another hospital	5	(1.7)	6	(2.0)	
Nursing facilities	1	(0.3)	1	(0.3)	
Postacute care	1	(0.3)	0	(0)	
Others	1	(0.3)	3	(1.0)	
**Vital signs 24 hours prior to discharge**					
Systolic BP (mmHg)	127.34	(19.70)	130.10	(19.42)	0.068
Systolic BP ≥ 100 mmHg (%)	282	(94.0)	293	(97.7)	0.025
Diastolic BP (mmHg), mean (SD)	73.24	(13.44)	74.96	(14.18)	0.102
Pulse rate (/min), mean (SD)	83.26	(14.18)	80.39	(12.92)	0.008
Temperature (°C), mean (SD)	36.5	(0.49)	36.44	(0.45)	0.087
Temperature ≥ 37.5°C, yes (%)	7	(2.3)	4	(1.3)	0.361
Respiratory rate (/min), mean (SD)	18.41	(2.29)	18.08	(1.79)	0.030
**Discharged medications**					
Number of categories, mean (SD)	6.99	(4.36)	5.60	(3.99)	<0.001
Number of total tablets, mean (SD)	77.14	(56.00)	63.26	(60.52)	0.002
Number of daily tablets, mean (SD)	11.27	(7.64)	9.27	(6.83)	<0.001
**Length of stay (days)**, mean (SD)	11.24	13.13	9.14	10.17	0.005
**Discharged conditions**					
With pressure injury (injuries), yes (%)	70	(23.3)	44	(14.7)	0.004
With a nasogastric tube, yes (%)	43	(14.3)	34	(11.3)	0.272
With a Foley catheter, yes (%)	26	(8.7)	19	(6.3)	0.278
With a trachea tube, yes (%)	6	(2.0)	4	(1.3)	0.524
With other catheter (s), yes (%)	121	(40.3)	104	(34.7)	0.152
Number of catheters, mean (SD)	0.80	(0.06)	0.64	(0.05)	0.034

### Independent association with early 14-day unplanned readmission

Six factors were significantly associated with readmission in the multivariable analysis (Table 5): previous admissions within 6 months (OR = 3.09; 95 % CI = 1.79–5.34), number of diagnoses in the past 1 year (OR = 1.07; 95 % CI = 1.01–1.13), MUST score (OR = 1.46; 95 % CI = 1.04–2.05), discharge with an NG tube (OR = 0.13; 95 % CI = 0.03–0.60), systolic blood pressure (OR = 0.98; 95 % CI = 0.97–0.99), and ear temperature within 24 h before discharge (OR = 2.49; 95 % CI = 1.34–4.64).

**Table 5:** Multivariate logistic regression of factors associated with early 14-day unplanned readmission

Variables	Adjusted odds ratio	95 % confidence interval	*p* value
Hospitalizations 6 months prior to the index admission	3.092	1.789–5.344	< 0.001
Number of diagnoses in past 1 year	1.069	1.011–1.131	0.019
Malnutrition Universal Screening Tool	1.458	1.037–2.047	0.030
Discharge with a nasogastric tube	0.125	0.026–0.597	0.009
Systolic blood pressure (mmHg)	0.980	0.965–0.995	0.010
Sex, female			0.749
Marital status, single			0.511
Religion, yes			0.196
Education, ≤elementary school			0.262
Area of residence, urban			0.235
Outpatient visits 6 months prior to the index admission			0.592
Emergency visits 6 months prior to the index admission			0.926
Charlson Comorbidity Index in past 1 year			0.697
Admission via emergency room			0.430
Mobility, independent			0.679
Bathing, independent			0.374
Mood (BSRS)			0.538
Urine incontinence, yes			0.503
Fall in the past 1 year, yes			0.366
White blood cell, ×10^3^ /UL			0.203
Hemoglobin, g/dL			0.906
Platelet, ×10^3^ /UL			0.109
Creatinine, mg/dL			0.608
Alanine aminotransferase, U/L			0.113
Potassium, mmol/L			0.185
Sodium, mmol/L			0.865
Discharge planning services, yes			0.241
Medical department of discharge			0.515
Physician’s experience (years)			0.846
Destination of discharge			0.408
Diastolic BP (mmHg)			0.808
Pulse rate (/min)			0.751
Respiratory rate (/min)			0.916
Categories of discharged medications			0.830
Total tablets of discharged medications			0.787
Length of stay (days)			0.693
Discharge with pressure injury (injuries), yes			0.371
Discharge with a Foley catheter, yes			0.663
Discharge with a trachea tube, yes			0.502
Discharge with other catheter (s), yes			0.184

The adjusted ORs indicated that patients who had a history of frequent hospitalizations in the past 6 months, multimorbidity, malnutrition at admission, no NG tube at discharge, and a lower systolic pressure and a higher ear temperature within 24 h of discharge were associated with an increased risk of 14-day unplanned readmission.

## Discussion

In this study, we found that predictors of early 14-day unplanned readmission were nonclinical with clinical factors presented at initial admission and clinical factors presented at discharge. To the best of our knowledge, our study is the first to comprehensively investigate factors related to baseline patient data, previous health care utilization, comorbidity, functional evaluation, laboratory data, and discharge to be potential risk factors simultaneously for early 14-day unplanned readmission in Taiwan.

A noteworthy finding of this study is that malnutrition at admission predicted early 14-day unplanned readmission, which is in agreement with recent studies in Singapore, Australia and Isarel [22–25]. Malnutrition is associated with adverse patient outcomes, and a standard policy in our hospital is to screen all patients using MUST at the time of admission. To date, few studies have included nutrition status as a potential factor in the study of predictors of unplanned readmission. Sharma et al. first noted that malnutrition at admission was a significant predictor of readmission in older patients [23] and later suggested that malnutrition, as also determined by MUST, was a strong predictor of early (0–7 days) and late (8–180 days) hospital readmission in adult patients in two tertiary hospital in Australia [22]. Our finding validates the results of Sharma et al. However, a recent study of unplanned 7-day readmission in pediatrics indicated that the relationship between malnutrition and risk of readmission may differ depending on the patient’s age [26]. In our study, we included adult patients and age was matched between the case and control group members; therefore, whether malnutrition as a factor predicting 14-day unplanned readmission differs by age group in Taiwan requires further investigation. Moreover, further research is needed to confirm the effect of improved nutrition status during hospitalization on unplanned readmissions.

We also found that patients who were discharged with an NG tube were less likely to have unplanned readmission compared with those discharged without an NG tube. In contrast to our finding, Wilmskoetter et al. noted that stroke patients with a percutaneous gastrostomy (PEG) feeding tube placed during their index hospital stay were twice as likely to be readmitted within 30 days compared with those without PEG tube placements [27]. In Taiwan, more than 90 % of patients who require enteral feeding choose an NG tube instead of a PEG tube because of the influence of cultural values [28], and little is known about the association between patients with an NG tube and unplanned readmission. One explanation could be that our hospital mandates all hospitalized patients with an NG tube to consult a homecare nurse. The consultation ensures a timely home visit after discharge, enabling regular NG tube changes at home. Home visits made by different clinical health care professionals have been shown to reduce unplanned admissions [29–31]; therefore, patients discharged with an NG tube had a lower risk of early14-day unplanned readmission in our study; this was probably a result of early home visit intervention. Future studies should include different home care service utilization as a potential variable to clarity the influence of home visits on early 14-day unplanned readmission.

Our finding that hospitalizations in the past 6 months is associated with 14-day unplanned readmission agrees with those of previous studies based on 28-day and 30-day unplanned readmission [32–36]. Shadmi et al. used data from before the index admission for early high-risk case identification and developed a 30-day readmission prediction model with high discriminative power compared with previously reported models that included only data from the time of discharge [36]. Prior ED attendance has been reported to be an independent predictor of 30-day unplanned readmission [34, 37], and Saleh et al. showed that ED visits in the past 1 year is an independent predictor for early 7-day readmission;[15] however, our results did not support this finding.

Our finding that early 14-day unplanned readmission is associated with multimorbidity in the past 1 year is in line with those of previous studies on 28-day and 30-day unplanned readmission [38, 39]. Although CCI score has been identified as a significant predictor in French, Australian, American, and Canadian studies, our analyses demonstrated significantly higher CCI scores in cases (*p*< 0.001) in univariate analyses but not in multivariable analyses [15, 32, 34, 37]. The possible effects of multicollinearity between CCI and other variables were assessed by confirming that the variance inflation factor did not exceed 4 for CCI and other variables in the multivariable analyses [40]. Type of index admission and length of stay have been identified as predictors of unplanned readmission and have been used in many prediction models for 30-day unplanned medication [15, 32, 34, 37, 41, 42], but these two factors were not independently associated with 14-day unplanned readmission in the present study, although univariate analyses showed significant differences between the cases and controls (*p* < 0.001 and *p* = 0.005, respectively).

Our finding that vital signs including systolic blood pressure and ear temperature within 24 h before discharge are associated with early 14-day unplanned readmission supports similar findings reported in previous studies on 30-day readmission. Sudhakar et al. demonstrated that higher systolic blood pressure is negatively associated with readmission in patients with heart failure at a tertiary hospital, which is in agreement with our study finding [43]. Saleh et al. pointed out that ≥ 1 vital sign instability at discharge is associated with both 30-day and early 7-day readmission [15]. Notably, research has suggested that characteristics at discharge are more predictive of early 7-day readmission [15]. Nevertheless, our finding that unstable vital signs at discharge are associated with an increased risk of unplanned readmission is consistent with previous studies suggesting that early readmissions are more likely to be related to clinical stability on discharge than 30-day readmission [8, 13, 14].

Prescription drug–related readmissions represent a nonnegligible proportion of readmissions, particularly among older patients [44–47]. In the study of Morandi et al., elderly patients with seven or more drug prescriptions were more likely to be readmitted to a rehabilitation hospital than elderly patients with fewer prescriptions were [47]. In our study, we included all adult patients and did not determine a particular number of discharge medication categories significantly associated with unplanned 14-day readmission in the multivariable analysis. Schwab et al. identified the prescription of nervous system drugs at discharge, including antidepressants, as a risk factor for avoidable readmission in patients aged over 75 years [44]. Our study did not include high-risk medications as a predictor variable, and future investigation is warranted to elucidate the effects of different medications on 14-day readmission risk in Taiwan.

 Our findings provide implications for health care providers and administrators designing systems to improve quality of care. Our study results will enable clinicians to identify patients at a high risk of hospital readmission and accordingly initiate interventions during hospitalization, for example, providing adequate information to patients and families with patients with frequent previous hospitalizations, multiple diseases, and malnutrition. Evidence also suggests that early nutrition intervention may help improve the nutrition status of malnourished patients, but whether such intervention can reduce unplanned readmission remains inconclusive. However, this study also revealed that predischarge interventions may be required for patients with clinical instability at discharge; such interventions include assessment of patient needs, arrangement of early outpatient follow-up, and referrals to health care resources in communities, such as home care services.

This study has several limitations. First, data collection was based on retrospective medical record extraction, which may have provided an inadequate report of all risk factors for readmission. Second, because of the case–control study design, risk factors significantly influenced by the matching criteria could not be evaluated or were potentially underestimated (age and disease-related group at index admission). Moreover, the changes in staffing, facilities, clinical practice, and referral patterns over the 1-year study period may have introduced unintended bias to the study results. However, the effect of these factors was reduced by propensity-score matching on admission to minimize seasonal variation. Third, we did not consider readmission to another facility because the information in the data set was limited to readmissions to the same hospital. Fourthly, we did not consider the effects of high-risk medications on discharge and major procedures performed on admission in the study, which may have confounded the results. Further investigations with access to large volumes of patient records including major therapeutic events, iatrogenic factors, and home care service utilization are warranted. Finally, this study was conducted among patients from a single academic tertiary hospital, and our findings may not be generalizable to patients in other facilities; hence, further external validation is required. Nonetheless, our study results could pave the way for future studies to understand factors associated with early 14-day hospital unplanned readmissions.

## Conclusions

Early unplanned readmissions are a major cause of health care burden, and timely identification of at-risk patients can help initiate effective interventions for reducing cost and improving quality of care. This case–control study of adult patients in a tertiary hospital in Taiwan revealed that frequent hospitalizations prior to admission, multimorbidity, malnutrition, the absence of an NG tube, and clinical instability upon discharge were associated with an increased risk of 14-day unplanned readmission. Additional studies are required to improve the prediction model of 14-day unplanned readmission risks and develop targeted interventions for high-risk patients.

## Data Availability

Data and resources will be shared with other eligible investigators through academically established means. The datasets used and analyzed during the study will be available from the corresponding author on reasonable request.
